# Paramedics' and pre-hospital physicians' assessments of anatomic injury in trauma patients: a cohort study

**DOI:** 10.1186/1757-7241-18-60

**Published:** 2010-11-22

**Authors:** Hetti Kirves, Lauri Handolin, Mika Niemelä, Janne Pitkäniemi, Tarja Randell

**Affiliations:** 1 Department of Anaesthesiology and Intensive Care Medicine, Helsinki University Central Hospital, Helsinki, Finland; 2 Department of Traumatology, Helsinki University Central Hospital, Helsinki, Finland; 3 Department of Neurosurgery, Helsinki University Central Hospital, Helsinki, Finland; 4 Hjelt institute, Department of Public Health, University of Helsinki, Helsinki, Finland

## Abstract

**Background:**

The pre-hospital assessment of a blunt trauma is difficult. Common triage tools are the mechanism of injury (MOI), vital signs, and anatomic injury (AI). Compared to the other tools, the clinical assessment of anatomic injury is more subjective than the others, and, hence, more dependent on the skills of the personnel.

The aim of the study was to estimate whether the training and qualifications of the personnel are associated with the accuracy of prediction of anatomic injury and the completion of pre-hospital procedures indicated by local guidelines.

**Methods:**

Adult trauma patients met by a trauma team at Helsinki University Trauma Centre during a 12-month period (n = 422) were retrospectively analysed. To evaluate the accuracy of prediction of anatomic injury, clinically assessed pre-hospital injuries in six body regions were compared to injuries assessed at hospital in two patient groups, the patients treated by pre-hospital physicians (group 1, n = 230) and those treated by paramedics (group 2, n = 190).

**Results:**

The groups were comparable in respect to age, sex, and MOI, but the patients treated by physicians were more severely injured than those treated by paramedics [ISS median (interquartile range) 16 (6-26) vs. 6 (2-10)], thus rendering direct comparison of the groups ineligible. The positive predictive values (95% confidence interval) of assessed injury were highest in head injury [0,91 (0,84-0,95) in group 1 and 0,86 (0,77-0,92) in group 2]. The negative predictive values were highest in abdominal injury [0,85 (0,79-0,89) in group 1 and 0,90 (0,84-0,93) in group 2]. The measurements of agreement between injuries assessed pre- and in-hospitally were moderate in thoracic and extremity injuries. Substantial kappa values (95% confidence interval) were achieved in head injury, 0,67 (0,57-0,77) in group 1 and 0,63 (0,52-0,74) in group 2. The rate of performing the pre-hospital procedures as indicated by the local instructions was 95-99%, except for decompression of tension pneumothorax.

**Conclusion:**

Accurate prediction of anatomic injury is challenging. No conclusive differences were seen in the ability of pre-hospital physicians and paramedics to predict anatomic injury in the respective patient populations.

## Background

Care provided in a specialized trauma centre has been shown to decrease mortality and to improve functional outcome after major trauma [1,2]. Therefore, pre-hospital identification of severe trauma is desirable to guarantee direct transport to a trauma centre. However, over-triage of severe injury should be minimized in order to avoid unnecessary burden to these facilities. Direct transport to a facility capable of definitive treatment is generally advisable also in minor and moderate injuries, as it can reduce the treatment delay and need for secondary transports.

A wide range of triage systems is presently available to distinguish between severe and less severe trauma, but, still, the assessment of blunt trauma in the pre-hospital setting is notoriously difficult. The mechanism of injury alone is generally a poor prognostic tool [3]. Physiologic criteria - at least the three core parameters, respiratory rate, systolic blood pressure, and Glasgow Coma Scale (GCS) scores - can predict mortality and the need of interventions [4-7]. Anatomic criteria can be useful, but they are not straight-forward. Studies on the activation criteria of trauma teams have shown that at least penetrating truncal injury, paralysis, and large (> 20% body surface area) burn injuries are associated with a need for urgent intervention, admission to an intensive care unit or death at the emergency department [8,9].

The results regarding the influence of the training and qualifications of the pre-hospital personnel on patient outcome are conflicting. Paramedics have been shown to be able to identify major trauma with high sensitivity but low specificity, but unable to assess the severity of injuries in individual body regions [10]. Anaesthesiologists perform a more precise field triage than paramedics [11], and pre-hospital physicians may even improve the survival of trauma patients [12,13]. On the other hand, implementation of a program of advanced life support (ALS) performed by paramedics did not improve the survival of trauma patients [14]. The importance of pre-hospital procedures in trauma care remains debated [15]. In addition to the clinical condition and the general setting (urban vs. rural), the rationale of performing pre-hospital procedures may largely depend on the experience and education of the personnel performing the procedures.

The effect of the training and qualifications of the personnel on the accuracy of prediction of anatomic injury has not been thoroughly investigated. The primary aim of the present study was to find out whether the paramedics and pre-hospital physicians were able to predict equally the anatomic injury. The secondary objectives were to evaluate (1) whether the pre-hospital procedures were completed as indicated by the local instructions, and (2) whether the ability to follow the instructions was related to the training and qualifications of the personnel.

## Methods

Helsinki University Central Hospital (HUCH) serves a population of ca. 1,5 million people in southern Finland. The Trauma Centre of HUCH, Töölö hospital, provides acute trauma care to a vast majority of major blunt trauma patients; pediatric patients without suspected brain injury and penetrating torso trauma patients are referred to other HUCH hospitals. The catchment area of Töölö hospital is served by various EMS providers organized in two EMS systems. The city of Helsinki has a single three-tiered EMS including one physician-staffed ground unit. The area surrounding Helsinki is served by some 50 local EMS providers and one physician-staffed unit equipped with an emergency medical helicopter and a ground unit. The training of EMS personnel varies ranging from rescuers with less than six months of training in emergency medicine to paramedics with four years of studies in university of applied sciences. Theoretically, the qualifications of EMS personnel are quite uniform, as the criteria for being competent to work in basic- and advanced-level ambulances are similar throughout the area, but the protocols to evaluate the skills vary. In contrast, the training and qualifications of EMS physicians are relatively uniform. All are specialists in anaesthesia and intensive care, some specialists thoroughly familiar with anaesthesia, or residents with several years of training; the mean amount of experience in EMS approaches ten years.

As the EMS systems in the catchment area of Töölö hospital are heterogeneous, the trauma guidelines are not uniform throughout the area. However, a few key issues regarding suspected major trauma are common in the local instructions: 1) tension pneumothorax should be decompressed, 2) patients with GCS 8 or less should have the airway secured, 3) intravenous (iv) line should be inserted if possible, 4) pre-notification must be given to the receiving hospital. If the units on scene are not capable of performing these procedures, they are supposed to ask for reinforcements.

The present study was an observational retrospective cohort study approved by the Ethical Committee of HUCH. Adult patients treated by a trauma team at Töölö hospital during a 12-month period in 2006 were analysed. The trauma team activation criteria were systolic blood pressure less than 90 mmHg; compromised airway/ventilation; GCS < 12; or mechanism of injury as defined by American College of Surgeons Committee on Trauma (ACS-COT) [16]. Inter-hospital transfers (referrals) were excluded. The pre-hospital electronic patient records and paper records were analysed, and all signs and symptoms documented in six different anatomic body regions (thorax, abdomen, pelvis, head, spine, extremities) were recorded; all procedures performed during the pre-hospital phase were also recorded. The injuries detected after arrival to hospital by physical examination, by imaging techniques, or during operation or in autopsy were aggregated. The injuries were classified using the Abbreviated Injury Scale (version 2005), AIS http://www.aams.org, for obtaining the Injury Severity Score, ISS [17].

For statistical analysis, SPSS 13.0 for Mac OS X was used. The categorical data were tested using the chi-square (Fisher's exact) test and the continuous data using the Mann-Whitney U test. The pre-hospital and in-hospital injuries were dichotomized so that any documented sign or symptom of injury in a given body region was defined as assessed injury. For example, bruised thorax or suspected quiet breathing sound mentioned in the pre-hospital patient documents would be interpreted as assessed injury and deemed as true positive if any injury in the thorax, such as thoracic contusion or pneumothorax, was later diagnosed at hospital. To determine the ability of pre-hospital assessed injuries to predict in-hospital assessed injuries, positive predictive values (PPVs) and negative predictive values (NPVs) were calculated in both patient groups. An interrater reliability analysis using the Kappa statistics was performed to determine consistency among pre-hospital and in-hospital raters [18]. In general, kappa is used in assessing the degree to which two raters, assessing the same cases, agree in regard to sorting the cases into categories. As presented by Landis et al., we consider kappa from 0.40 to 0.59 to indicate moderate, from 0.60 to 0.79 substantial, and 0.80 outstanding level of agreement [18], even though a stricter classification has been proposed by Shrout[19]. In the present study, the in-hospital raters - having access to all possible means to diagnose injury - are considered correct; thus, the higher the kappa values are, the better the pre-hospital raters perform.

## Results

A total of 422 patients were identified for analysis. Pre-hospital physicians had participated in the treatment of 230 patients (group 1), and 190 patients were treated by paramedics alone (group 2). In two cases, the presence or absence of a physician remained uncertain resulting in 420 included patients for the analysis comparing the two groups. The patient characteristics, MOI, and pre-hospital time intervals are presented in Table 1, and the physiological variables in Table 2. The patients treated by the pre-hospital physicians (group 1) were more severely injured and had higher injury severity scores (ISS) than those treated by the paramedics (group 2) (Table 1). Concomitantly, the patients in group 1 were more often comatose and tended to have lower systolic blood pressures than the patients in group 2 (Table 2).

**Table 1:** Patient characteristics.

Characteristics (unit)	Missing data (n)	All patients (n = 422, 100%)	Group 1: Patients treated by physicians (n = 230, 55%)	Group 2: Patients treated by paramedics (n = 190, 45%)	p-value groups 1 and 2 compared
**Age (years)**	0	37 (25-52)	37 (25-51)	38 (27-52)	0,704
**Sex (M/F) [%]**	0	76,7/23,3	77,0/23,0	76,3/23,7	0,908
**Mechanism of injury (%)**	0				
**1. motor vehicle accident**		44,8 (40,1-49,6)	40,9 (34,7-47,3)	49,5 (42,4-56,5)	0,094
**2. motorcycle**		12,6 (9,7-16,1)	10,9 (7,5-15,6)	14,7 (10,4-20,5)	0,242
**3. cyclist**		3,6 (2,2-5,8)	3,9 (2,1-7,3)	3,2 (1,5-6,7)	0,795
**4. pedestrian**		9,8 (7,3-12,9)	10,9 (7,5-15,6)	8,4 (5,3-13,2)	0,415
**5. fall > 4m**		15,5 (12,2-19,2)	17,8 (13,4-23,3)	12,6 (8,7-18,1)	0,175
**6. fall < 4 m**		7,4 (5,2-10,2)	7,8 (5,0-12,0)	6,8 (4,1-11,4)	0,852
**7. stab or gun shot wound**		1,2 (0,5-2,7)	1,7 (0,7-4,4)	0,5 (0,1-2,9)	0,383
**8. other**		5,2 (3,5-7,8)	6,1 (3,7-10,0)	4,2 (2,2-8,1)	0,510
**Time intervals (min)**					
**to first responding unit**	62	10 (7-13)	10 (7-13)	10 (8-14)	nc
**on scene**	118	27 (21-36)	29 (22-40)	25 (20-32)	nc
**Call to hospital**	18	62 (49-78)	62 (48-78)	63 (52-77)	0,403
**ISS**	0	10 (4-18)	16 (6-26)	6 (2-10)	< 0,001

Median (25^th^-75^th ^percentile) for all non-categorical and frequency (%) (95% confidence interval) for all categorical variables. Two cases were not classified into either of the groups due to missing data. nc, not calculated because of missing values.

**Table 2:** Physiologic variables.

Characteristics (unit)	Missing data (n)	All patients (n = 422, 100%)	Group 1: Patients treated by physicians (n = 230, 55%)	Group 2: Patients treated by paramedics (n = 190, 45%)	p-value - groups 1 and 2 compared
**First spontaneous breathing** **rate (/min)**	110	16 (14-20)	16 (14-20)	18 (16-20)	nc
**First systolic blood pressure** **(mmHg)**	18	130 (113-140)	125 (110-140)	131 (120-145)	0,003
**First pulse rate (/min)**	5	90 (78-105)	90 (74-104)	92 (80-108)	0,089
**First GCS**	8	15 (12-15)	14 (6-15)	15 (15-15)	< 0,001
**First SpO_2 _(%)**	116	96 (94-98)	96 (91-97)	97 (95-98)	nc

Median (25^th^-75^th ^percentile) for all variables. Two cases were not classified into either of the groups due to missing data. nc, not calculated because of missing values.

To compare the ability of paramedics and pre-hospital physicians to predict anatomical injury in six body regions, positive and negative predictive values of documented signs and symptoms were calculated in both groups separately (Table 3). In the six body regions studied, only one marginal difference in the predictive values was detected: in spinal injury, the NPV of assessed injury in group 1 was lower than in group 2. In general, there was a trend towards higher PPVs in group 1, accompanied with higher NPVs in group 2.

**Table 3:** Pre- and in-hospital assessed injuries and predictive values in the two patient populations.

	Group 1: Patients treated by pre-hospital physician (n = 230, 55%)				Group 2: Patients treated by paramedics (n = 190, 45%)			
Body region	assessed injury: pre/in-hospital		Positive predictive value	Negative predictive value	assessed injury: pre/in-hospital		Positive predictive value	Negative predictive value
						
	Pre	In			Pre	In		
**Thx**	30,0	53,0	0,90 (0,81-0,95)	0,63 (0,55-0,70)	42,1	46,3	0,73 (0,62-0,81)	0,73 (0,64-0,80)
**Abd**.	13,0	18,7	0,43 (0,27-0,61)	0,85 (0,79-0,89)	14,2	12,6	0,26 (0,13-0,45)	0,90 (0,84-0,93)
**Pelvis**	15,2	25,7	0,69 (0,52-0,81)	0,82 (0,76-0,87)	12,6	14,2	0,42 (0,25-0,61)	0,90 (0,84-0,94)
**Head**	64,3	67,8	0,91 (0,86-0,95)	0,74 (0,64-0,83)	48,4	51,6	0,80 (0,71-0,87)	0,83 (0,74-0,89)
**Spine**	20,4	34,8	0,62 (0,47-0,74)	0,72 (0,62-0,78)	33,7	23,7	0,42 (0,31-0,54)	0,86 (0,79-0,91)
**Extr**.	47,0	65,2	0,91 (0,84-0,95)	0,57 (0,49-0,66)	48,4	57,4	0,86 (0,77-0,92)	0,69 (0,60-0,78)

Frequency (%) of assessed injuries and predictive value (95% confidence interval). Thx, thorax; Abd., abdomen; extr., extremities

The measurement of agreement between pre-hospital and in-hospital assessed injuries in the six body regions was estimated using kappa statistics (Table 4). In the two groups, the kappa values were comparable in all the studied body regions. Only in head injury, the agreement between the pre-hospital and in-hospital raters was substantial. In injuries to extremities or thorax, a moderate agreement between the raters was achieved; in abdominal, pelvic, and spinal injury, the agreement was even lower (Table 4).

**Table 4:** Measurement of agreement between pre-hospital and in-hospital assessed injuries in the two patient populations.

Body region	Group 1: Patients treated by pre-hospital physician (n = 230, 55%)	Group 2: Patients treated by paramedics (n = 190, 45%)
**Thorax**	0,43 (0,33-0,53)	0,45 (0,32-0,57)
**Abdomen**	0,24 (0,084-0,39)	0,16 (-0,014-0,35)
**Pelvis**	0,40 (0,26-0,53)	0,30 (0,11-0,48)
**Head**	0,67 (0,57-0,77)	0,63 (0,52-0,74)
**Spine**	0,27 (0,14-0,40)	0,30 (0,16-0,44)
**Extremities**	0,47 (0,37-0,58)	0,55 (0,43-0,67)

Kappa value (95% confidence interval). Kappa from 0.40 to 0.59 are considered moderate, 0.60 to 0.79 substantial, and 0.80 outstanding.

The instructions regarding procedures in major trauma were followed meticulously (Table 5). The pre-notification call to the receiving hospital was neglected only in three cases (0,71%). Securing the airway in suspected severe traumatic brain injury was performed dutifully. Seventy-nine patients (19%) arrived to ED with GCS 8 or less and only four of them (5,1%) arrived to hospital without a secured airway. Intravenous line was absent in 15 patients (3,6%), 11 in the group treated by the paramedics. Tension pneumothorax was diagnosed in 14 patients in the pre-hospital phase or in the emergency department (ED). In nine of these patients, decompression of tension pneumothorax was not attempted before arrival to ED, suggesting that the procedure had not been considered in the pre-hospital phase, or, alternatively, tension developed after arrival to ED. The five patients who had the tension pneumothorax decompressed before arrival to ED were all met by a pre-hospital physician. In addition, in two cases, decompression was attempted, but tension was detected neither during the procedure nor later in the ED.

**Table 5:** Traumatic conditions warranting pre-hospital interventions and procedures omitted in the pre-hospital phase.

Characteristics (unit)	All patients (n = 422, 100%)	Group 1: Patients treated by physicians (n = 230, 55%)	Group 2: Patients treated by paramedics (n = 190, 45%)
**Pre-notification not given to hospital (n)**	3	1	2

**Tension pneumothorax (n)**	14	11	3
**- decompression not attempted in the pre-hospital phase (n)**	9	6	3

**Patients arriving hospital with GCS 8 or less (n)**	79	76	3
**- airway not secured (n)**	4	1	3

**Patients arriving hospital without iv line (n)**	15	4	11

## Discussion

To our knowledge, the present study is the first to compare the ability of pre-hospital physicians and paramedics to predict anatomic injury. Presumably, particularly in the successful detection of concealed injury, experience and education of the personnel could play a significant role. In the study by Rehn et al., pre-hospital anaesthesiologists performed more correct triage than paramedics using the anatomic trauma-team activation criteria as a whole, but the usage of the distinct criteria was not analyzed [11].

In the present study, pre-hospital identification of an injury in exposed body parts, extremities and head, was, as expected, the most accurate. Identification of a thoracic injury was less accurate; in abdominal, pelvic, and spinal injuries the consistency of pre-hospital assessed injury with in-hospital assessed injury was poor. Regarding the ISS and GCS scores and systolic blood pressure, the patients met by pre-hospital physicians were more severely injured than those met by paramedics only. The predictive values of the pre-hospital assessment of injury as well as the kappa measurements of agreement between the pre- and in-hospital raters seemed comparable regardless the training and qualifications of the pre-hospital personnel in the respective patient populations. To conclude, the ability of paramedics and pre-hospital physicians to find and document injuries in the patient populations they meet did not significantly differ from each other.

As the patients treated by pre-hospital physicians were more severely injured than those treated by paramedics, the risk of a false positive finding was much smaller for a physician than for a paramedic. Therefore, the trend towards higher PPV in the patient group treated by physicians and higher NPV in that treated by paramedics is understandable. In clinical decision-making, at least in systems resembling the present one, it might be helpful to keep this trend in mind: if a patient is escorted to hospital by a pre-hospital physician and the physician suspects an injury in a given body region, he may be right; if the patient is escorted by paramedics only and the paramedics claim that the patient does *not *have an injury in a given body region, they may be right. The lack of a conclusive difference in the overall diagnostic performance of paramedics and pre-hospital physicians is particularly interesting, as, in the EMS systems studied, the pre-hospital physicians are highly specialized and experienced professionals, whereas the background and experience of the paramedics can vary. This should not be interpreted as futility of education and training; rather, the deduction should be that the allocation of resources by the dispatch centre is well targeted, and the skills of the personnel groups are comparable in their respective patient populations.

The overall rate of performing procedures in the pre-hospital phase as indicated by the local instructions was high, 95-99%, for insertion of venous access and securing the airway in suspected severe traumatic brain injury, as well as for timely pre-notification to the receiving hospital. The high pre-notification rate is in contrast to previously reported results from the United Kingdom [20], where the receiving hospital was not pre-notified for a majority of severely injured patients (ISS >15). In the system studied, ambulance crews are instructed to call the pre-notification with a very low threshold; as a result, only approximately 60% of these pre-notification calls lead to trauma team activation (TTA) [21]. Further, 65% of these TTAs are futile if only major trauma as defined by ISS > 15 is deemed worth TTA. This straightforward definition of major trauma is possibly oversimplified for practical purposes; there are patients who might benefit from TTA even if they fail to reach the ISS cut-off point [22].

The decompression of tension pneumothorax made an exception to the meticulous adherence to the local procedural instructions. The diagnosis is not always self-evident in the pre-hospital setting; decompression is recommended if clinical suspicion is raised [23]. As the process of formation of tension within a pneumothorax is dynamic, it is possible that in some cases clinically significant tension had not yet developed before arrival to hospital, and, thus, the pre-hospital intervention would have been unnecessary. According to the local instructions, if the units on scene are not capable of performing the procedures instructed, they should call reinforcements. However, in some cases in the close vicinity of the receiving hospital, it can be logical to transport the patient to the hospital without delay. This may partially explain the tendency of omitted procedures to accumulate in the patient group treated by paramedics. Alternatively, the ability to recognize the condition justifying the procedures could be the limiting factor.

The present study has considerable limitations. Some patients may have been missed, and missing information in general presents considerable problems. The documentation requirements are the same for physicians and paramedics, but we cannot rule out the possibility that physicians do better documentation than paramedics - or vice versa. During the study period, there was no uniform policy of quantitative assessment of suspected severity of injury in pre-hospital documentation; thus, crude dichotomy in respect of detecting injury had to be used, possibly distorting the results even further. In addition, there are risks of review bias to be considered. Even though we estimate the likelihood of diagnostic review bias quite low, the possibility cannot be completely ruled out, since the interpretation of the imaging results may, in some cases, be affected by the knowledge of the radiologist of pre-hospital assessed injury. Incorporation bias is also possible, as, in some cases, the pre-hospital assessed injury may have been used to establish the final diagnosis; however, significant incorporation bias is unlikely, as the pre-hospital assessed injuries have to be verified at hospital to justify documenting in the diagnoses or status section of the patient documents. Most importantly, as the study was observational, the formation of the patient groups was not random. The pre-hospital physician could have been omitted from the emergency response for several reasons: the emergency call was not rated to the highest risk group, and the physician-manned unit was not dispatched; the physician-manned unit was dispatched, but on-scene information from the first responding units led to cancellation of the mission; or the pre-hospital physician was not available due to logistic reasons or coincident high-risk mission. This non-random formation of groups led to profound bias regarding the injury severity. Thus, the patient groups cannot be directly compared, and all interpretations are to be made with great caution.

## Conclusions

In the present study, pre-hospital physicians met patients suffering from more severe injuries than patients met by paramedics only. We were unable to detect any conclusive difference in the ability of paramedics and pre-hospital physicians to predict anatomic injury in the patient populations they met. Prospective studies in systems large enough to allow stratification required to address the skewed injury severity pattern would clarify the issue.

## Competing interests

The authors declare that they have no competing interests.

## Authors' contributions

HK designed the study, collected, and analysed the data as well as wrote the draft of the manuscript. JP adviced and helped in the statistical analysis and interpretation of the data. LH, MN, and TR helped in the study design and participated in the drafting and finalizing the manuscript. All the authors have read and approved the final manuscript.
